# High-resolution global maps of tidal flat ecosystems from 1984 to 2019

**DOI:** 10.1038/s41597-022-01635-5

**Published:** 2022-09-06

**Authors:** Nicholas J. Murray, Stuart P. Phinn, Richard A. Fuller, Michael DeWitt, Renata Ferrari, Renee Johnston, Nicholas Clinton, Mitchell B. Lyons

**Affiliations:** 1 College of Science and Engineering, James Cook University, Townsville, Queensland Australia; 2 Remote Sensing Research Centre, School of Earth and Environmental Sciences, The University of Queensland, St Lucia, Queensland 4072 Australia; 3 School of Biological Sciences, The University of Queensland, St Lucia, QLD Australia; 4 Google Inc., 1600 Amphitheater Parkway, Mountain View, CA 94043 USA; 5 Australian Institute of Marine Science, Townsville, 4810 Australia; 6 Centre for Ecosystem Science, School of Biological, Earth and Environmental Science, University of New South Wales, Sydney, Australia

**Keywords:** Macroecology, Ecosystem ecology, Marine biology

## Abstract

Assessments of the status of tidal flats, one of the most extensive coastal ecosystems, have been hampered by a lack of data on their global distribution and change. Here we present globally consistent, spatially-explicit data of the occurrence of tidal flats, defined as sand, rock or mud flats that undergo regular tidal inundation. More than 1.3 million Landsat images were processed to 54 composite metrics for twelve 3-year periods, spanning four decades (1984–1986 to 2017–2019). The composite metrics were used as predictor variables in a machine-learning classification trained with more than 10,000 globally distributed training samples. We assessed accuracy of the classification with 1,348 stratified random samples across the mapped area, which indicated overall map accuracies of 82.2% (80.0–84.3%, 95% confidence interval) and 86.1% (84.2–86.8%, 95% CI) for version 1.1 and 1.2 of the data, respectively. We expect these maps will provide a means to measure and monitor a range of processes that are affecting coastal ecosystems, including the impacts of human population growth and sea level rise.

## Abstract (summary)

**Table:** 

Measurement(s)	ecosystem occurrence
Technology Type(s)	earth observation
Sample Characteristic - Environment	tidal flats • coastal wetlands
Sample Characteristic - Location	global

## Background & Summary

Tidal flats are a globally widespread coastal ecosystem that occur at the interface between land and sea^1,2^. They occur at all latitudes in physical habitats that are low-sloping and influenced by tides, and include ecotypes ranging from tidal rock platforms to fine-grained silts and clays^1–4^. Increasing human populations along the global coastline have caused extensive loss, degradation and fragmentation of tidal flat ecosystems worldwide^1,5–8^. Yet, owing to difficulties in remote-sensing a widely distributed habitat type that is regularly obscured by water in each tidal cycle^1,4^, until recently there has been no resource sufficient for assessing the distribution and change of tidal flats at the global scale.

Satellite remote sensing has supported global analyses of the distribution and change of an increasing variety of land cover types^9^. Recent technological break-throughs in the use of distributed computing for analyses of earth observation data and improved accessibility to satellite data archives have permitted end-to-end global-scale analyses that were previously impossible to achieve^10–12^. High resolution spatial datasets are now freely available for features including croplands^13^, bare land^14^, shorelines^15^, ecological settings^16^, forest cover^14,17–19^, surface water^20–23^, mangroves^8,24–28^, tidal wetlands^8^, rivers and streams^29^, coral reefs^30,31^ and river ice^32^, transforming our ability to estimate planetary boundaries^33^, monitor human impacts to the environment^34^, develop global accounts of biodiversity^3^, and estimate risks to ecosystems^12,35^. With many of Earth’s ecosystems undergoing continued loss and degradation^3^, including coastal ecosystems^1,36–39^, there is a strong need to continue to expand the catalogue of high quality globally consistent datasets regarding the spatial distribution and temporal trends of earth’s ecosystems^3,12,35^.

Here we describe the global tidal flat dataset, developed as part of the Global Intertidal Change program (http://globalintertidalchange.org), which is a consistent, 30-m representation of the global distribution of tidal flats over a 35-year period (1984–2019). These data have already been used to report the global distribution and change of tidal flat ecosystems^1^, pressures they are subject to^1,40^, extent of protection^40^ and have supported local-scale analyses of loss and gain^41,42^. Additionally, they have supported syntheses of global wetland areas^3,43^, assessments of impacts to species^44^, efforts to compile the global distributions of habitat types^45^ and ecosystems^2,46^, model the habitat preferences of migratory shorebirds^47^, protected area planning^40^ and have been identified as a potential indicator for the post-2020 Convention on Biological Diversity global biodiversity framework^48^.

The dataset described here is available in two versions that address a trade-off between spatial coverage and length of time-series: (i) a long, 11-step, time-series of the global extent of tidal flats (1984–2016; version 1.1), and (ii) a new shorter, 7-step time-series with improved spatial coverage and an additional time-step (1999–2019; version 1.2)^49^.

## Methods

### Overview of methods

**Fig. 1:**
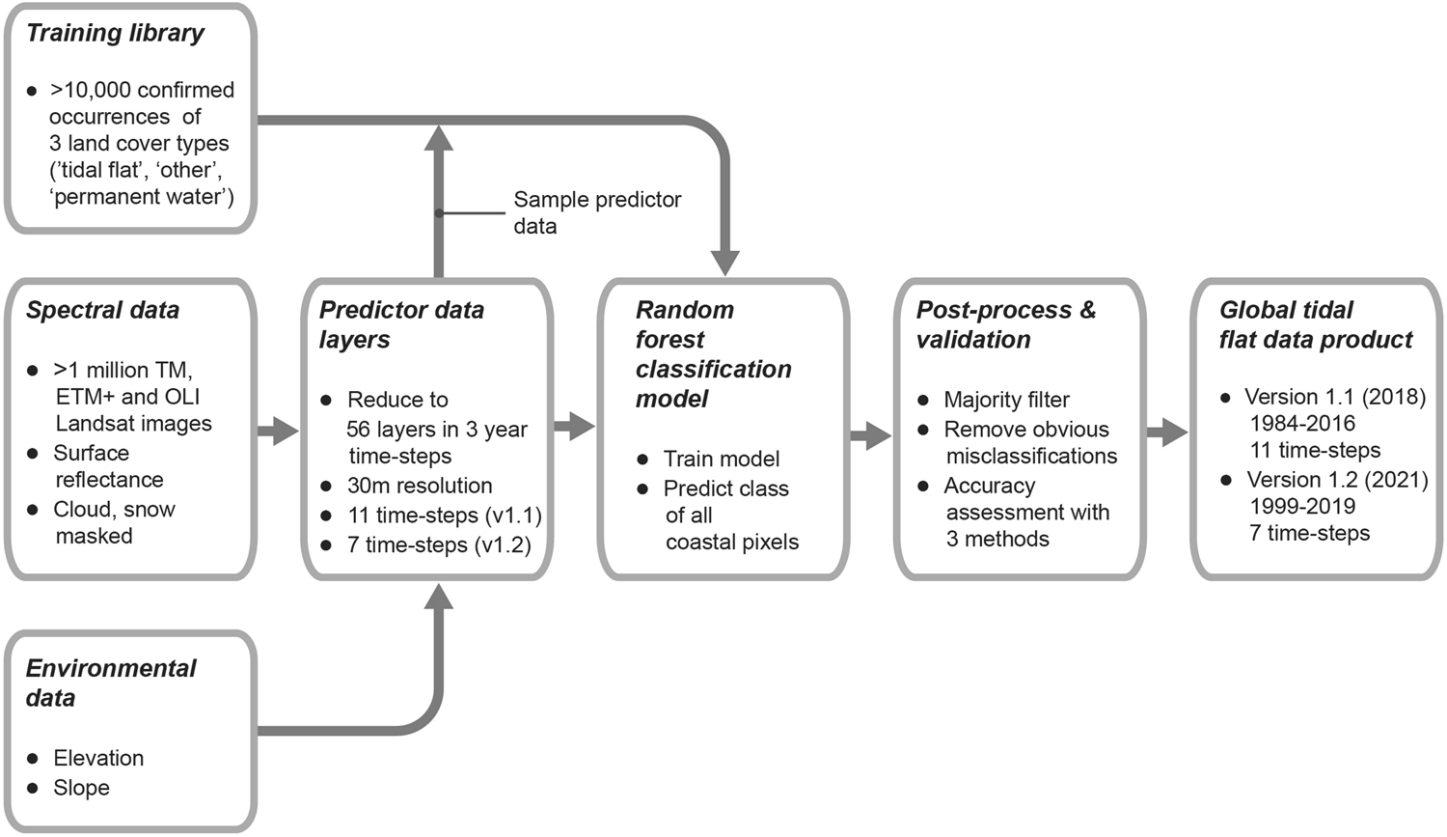
Simplified remote sensing classification approach for mapping the global distribution of tidal flats. Training data is used to develop a random forest model with 56 predictor data layers that assigns each pixel in the mapping area to one of three classes. The maps are then post-processed and delivered as map products representing the distribution of tidal flat ecosystems from 1984 to 2019.

These methods are expanded versions of descriptions in our related work^1^, following a generalised classification workflow summarised in Fig. 1. In particular, we focus here on describing our classification methods and detailing the methodological advances implemented to yield improved overall accuracy in the first major dataset update (version 1.2; 1999–2019).

The global tidal flat dataset was developed for the global shoreline between 60°N to 60°S from 1984 to 2019 (version 1.1, 1984–2016; version 1.2; 1999–2019)^49^. In this domain, the analysis was restricted to avoid elevated areas or deep water where tidal flats were not expected to occur to reduce the computing resources required to perform the full global analysis. Version 1.1^49^ was limited to the area where Landsat Archive scenes (1984–2019) intersected a 1-km buffer of the coastline, and further restricted to areas where tidal flat habitats were expected to occur, which we defined as terrestrial areas below 100-m elevation and within 5-km of the coast, and marine areas above the 100-m depth contour and within 50-km of the coast (using the Shuttle Radar Topography Mission and ETOPO1 Global Relief Model data)^1,50,51^.

The map area was modified for version 1.2 to reflect an improved knowledge of the distribution of coastal ecosystems^1,24,52^, allowing us to further reduce the total mapped area and associated computing resource use (see Usage Notes). The analysis was performed in the area above the 40-m depth contour, below 40-m elevation and within 5-km of the coast. To account for coastal ecosystems that may occur outside of this area^28^, we included the area within a 5-km buffer to all known spatial datasets depicting coastal wetlands^1,24,52,53^.

The binary raster file “datamask”, delivered with each version of the dataset, indicates all areas where the classification was implemented.

### Training data

**Fig. 2:**
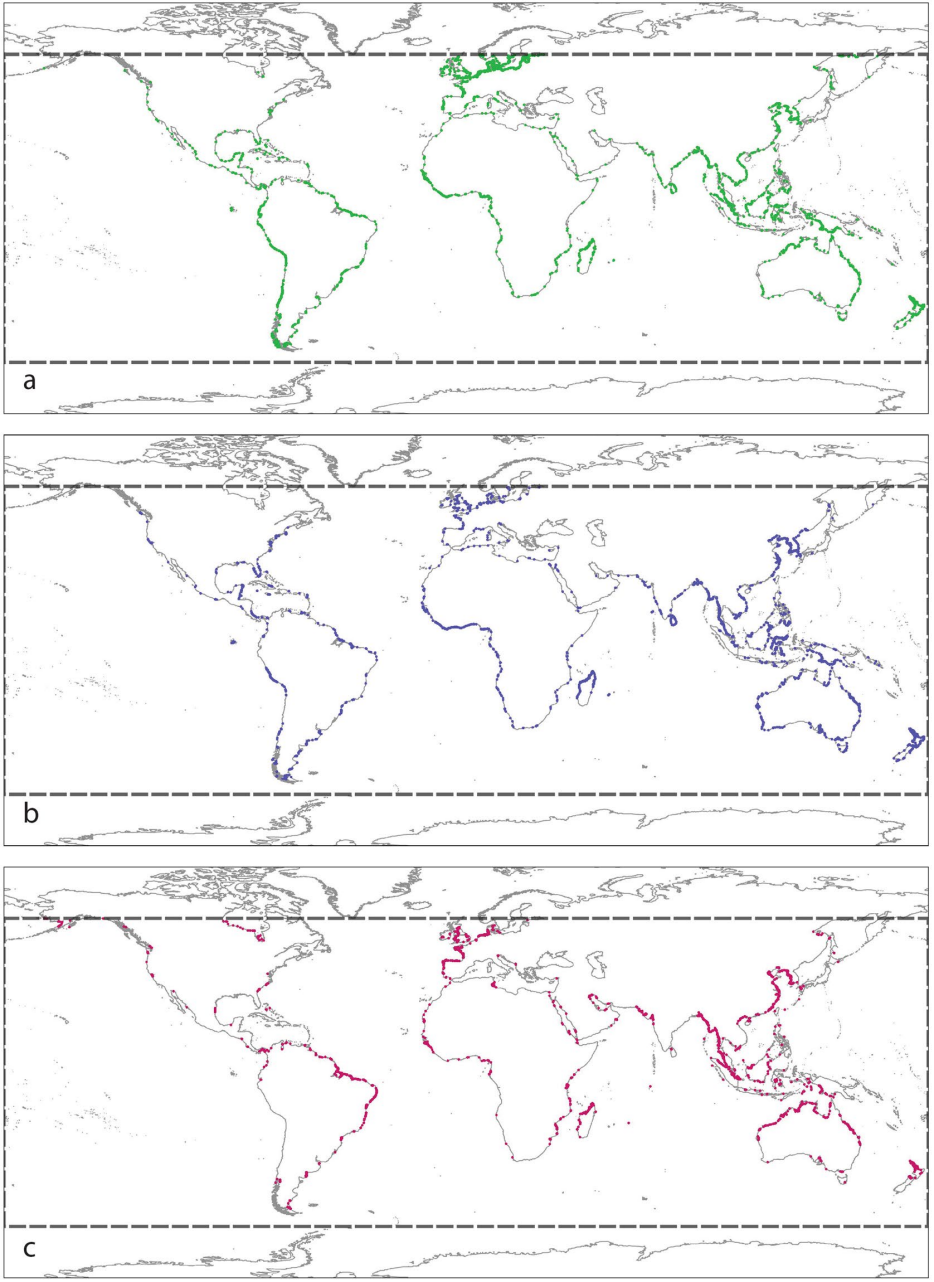
Global map showing the distribution of the training set used to map the global distribution of tidal flats between 60°N to 60°S. (**a**) Training set annotated with class ‘Other’. (**b**) Training set annotated with class ‘Permanent water’. (**c**) Training set annotated with class ‘Tidal flat’. The figure shows that training data was collected along the entire global coastline, though training samples for tidal flat are naturally concentrated in areas where tidal flats occur. The bounding box of the analysis is shown with a bold dashed line. Note maps show training data used for Version 1.2 of the tidal flat maps (n = 14,100 points).

**Fig. 3:**
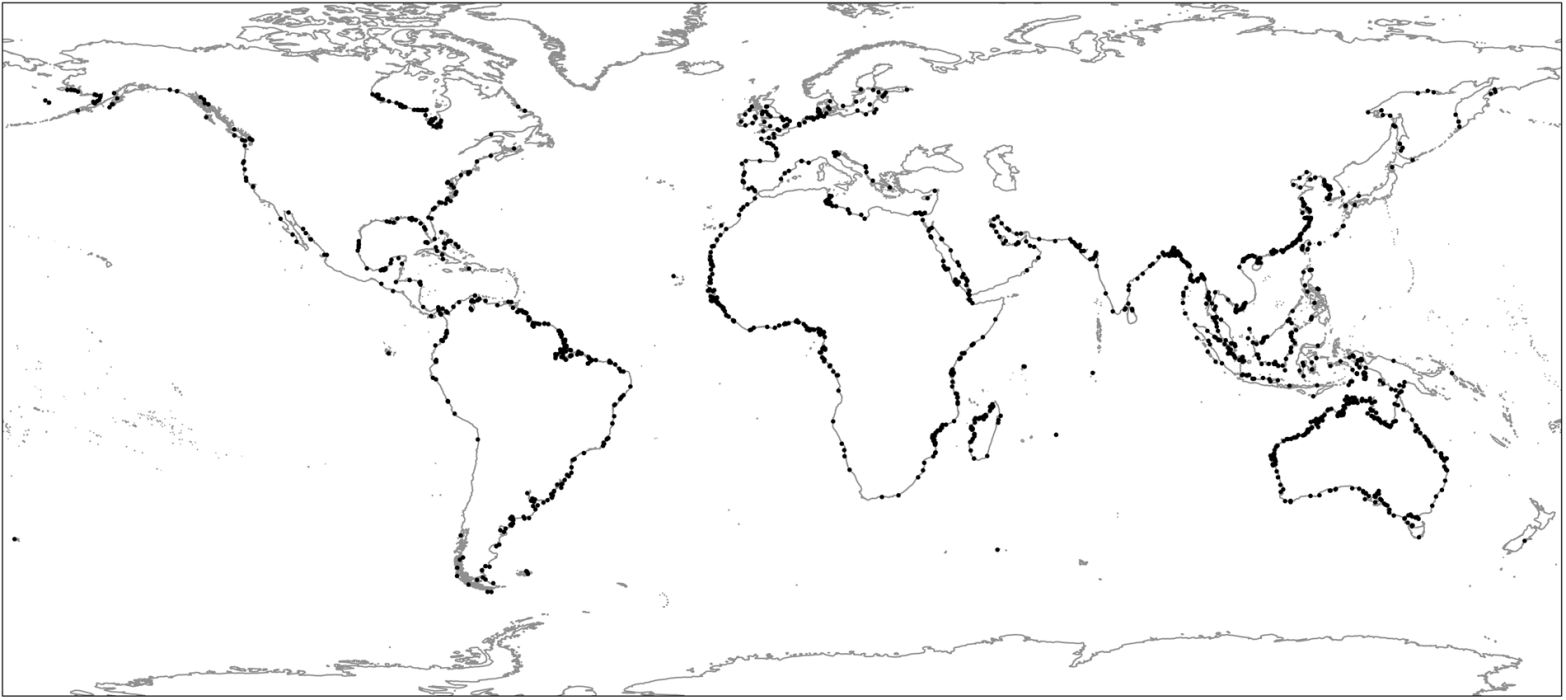
Distribution of the validation set used for the independent accuracy assessment of the global tidal flat dataset. The samples (n = 1,358) were assigned to the classes (‘tidal flat’ and ‘other’) by three independent analysts. The validation set was randomly sampled from the mapped area, stratified by class and continent. In addition to showing the global distribution of the validation samples, the figure highlights concentrations of validation samples in areas with greater extent of tidal flats, including the Australian coast, the northern coast of South America, and the Chinese coast. The bounding box of the analysis is shown with a bold dashed line. Figure sourced from^1^ and used to validate version 1.1 of the dataset.

To support the remote sensing classification model developed for mapping tidal flats, we established a globally distributed training set of confirmed locations of three target map classes (‘tidal flat’, ‘permanent water’, ‘other’; Fig. 2). To collect training samples for the training set, we used our extensive global field experience in tidal flat ecosystems, data and maps from peer-reviewed publications, and developed an online interactive application to enable simultaneous reference to six image sets to assist with confirming the location of tidal flats, including multiple visualisations of the Landsat satellite imagery in the validation period (2014–2016). The image sets were (1) high resolution Google Earth imagery, (2) the median of the Landsat Operational Land Imager (OLI) Near Infrared (NIR) band, (3) a Landsat OLI true colour composite (median values of bands 4, 3 and 2), (4) a Landsat OLI false colour image composite (median values of bands 5, 4 and 3), (5) the standard deviation of the Normalised Difference Water Index (NDWI), and (6) the standard deviation of the Automatic Water Extraction Index (AWEI). To further enable confirmed occurrences to be recorded in the training set, analysts could also refer to time-series of Google Earth imagery to explore tidal inundation dynamics at a sample location if required. A total of 10,701 geo-referenced points annotated with three target classes for classification were developed for version 1.1 (run in 2018), which was enlarged to 14,100 points for version 1.2 (run in 2021) using the same methods Figs. 1–3.

### Predictor variables

We developed a set of 56 predictor variables to be used in the remote sensing classification^1^. Our predictor set consisted of 54 spectral variables derived from all Landsat Archive imagery collected during the study period (1984–2019), and a further two static variables that represent the occurrence of surface water^20^ and topographic and bathymetric information from ETOPO1 Global Relief model^50^, which was resampled to 30-m. A total of 707,528 images were used for version 1.1 (1984–2016) and 1,166,385 for version 1.2 (1999–2019).

To enable time-series remote sensing classifications, we produced each spectral predictor data layer from stacks of pre-processed^54,55^ Landsat images that comprised all images acquired within each three-year time period across the time series (12 time-steps, 1984–2019, Table 1). Prior to assembling the image stacks for each time step, we masked pixels identified as cloud, cloud shadow and snow in each image by applying the FMask algorithm^56^. In addition to pixel masking, we added bands to each image that represented several Landsat indices, including Normalised Difference Water Index and the Automatic Water Extraction Index (see^1^). The image stacks were then reduced by band to per-pixel summary statistics, yielding 54 spectral predictor layers per time period for the classification model^1^.

**Table 1:** The number of Landsat images included in the remote sensing analysis of global tidal flats.

Time Step	Start	End	Version 1.1		Version 1.2	
			Mapped	No. Landsat images	Mapped	No. Landsat images
1	1984-01-01	1986-12-31	•	18,284	—	—
2	1987-01-01	1989-12-31	•	26,894	—	—
3	1990-01-01	1992-12-31	•	27,586	—	—
4	1993-01-01	1995-12-31	•	32,330	—	—
5	1996-01-01	1998-12-31	•	32,210	—	—
6	1999-01-01	2001-12-31	•	75,240	•	130,639
7	2002-01-01	2004-12-31	•	69,425	•	129,591
8	2005-01-01	2007-12-31	•	72,394	•	139,787
9	2008-01-01	2010-12-31	•	80,617	•	132,252
10	2011-01-01	2013-12-31	•	85,572	•	131,754
11	2014-01-01	2016-12-31	•	186,976	•	250,166
12	2017-01-01	2019-12-31	—	—	•	252,196
Total				707,528		1,166,385

Note that owing to an increased amount of Landsat data available in Google Earth Engine at the time of running the analyses, version 1.2 (2020) has greater coverage than version 1.1 (2018). Owing to resource limitations and to align with many other global map products, version 1.2 is produced only for the years 1999–2019.

### Remote sensing classification

We used the random forest algorithm^57^ to assign every 30-m pixel in the mapped area to the classes ‘tidal flat’, ‘permanent water’ or ‘other’. We trained the model using the training set annotated with pixel values for each of the 56 predictor variables, with the spectral data sampled from the 2014–2016 Landsat image stacks to align with the period for which training data was developed. We performed the global classifications in Google Earth Engine, with paramaterisations between version 1.1 and version 1.2 only differing only by the number of trees evaluated (ntree = 10, version 1.1; ntree = 100, version 1.2). We ran the analysis for each time-step in the spectral data, yielding maps of tidal flats for 11 time-steps (version 1.1) and 7 time-steps (version 1.2).

### Post processing

After performing the classification, we masked the permanent water and land classes, ran a majority filter to remove isolated pixels identified as tidal flat and manually removed obvious misclassifications. As part of the data package, we provide a set of quality assurance layers that represent per-pixel the depth of the image stacks used in the classification (“qa_pixel_count_20172019_V1_2”). The data ingestion, predictor compilation and classification model was implemented end-to-end in Google Earth Engine^10^.

## Data Records

The global tidal flat time-series maps version 1.1 (1984–2016) and version 1.2 (1999–2019)^49^ are made available directly via Google Earth Engine (https://earthengine.google.com/; Version 1.1 Asset ID: UQ/murray/Intertidal/v1_1/global_intertidal; Version 1.2 Asset ID: UQ/murray/Intertidal/v1_2/global_intertidal). Model training data and code are archived on Zenodo^58^. In addition, the data are made interactively available at the global intertidal change website (https://www.intertidal.app/download) and at the UNEP-WCMC Ocean Data Viewer (https://data.unep-wcmc.org/datasets/47). Additional data layers to interpret the global tidal flat data include a data mask depicting the mapped area (“datamask”) and a quality assurance layer representing the number of Landsat pixels used in the analysis (“qa_pixel_count”). To support data archiving required for this manuscript, version 1.1 and version 1.2 have been archived in a Figshare data repository^49^.

## Technical Validation

Validation of the version 1.1 global tidal flat data was performed in three ways, (1) a standard remote sensing error matrix approach^59^, (2) a bootstrapping approach to enable the estimation of overall map accuracy and confidence intervals^60^, and (3) an independent map agreement approach^1^. To support the first two approaches, we randomly sampled the mapped area, stratified by map class and continent, to form a validation set. The size of the validation set was determined using power analysis (n = 1,358 random samples) and later confirmed to be sufficient with post-hoc sensitivity analyses^1^. Validating a highly dynamic target map class at the global scale is a challenge. We therefore provided the set of validation points to three independent, experienced remote sensing analysts, who used the online image viewer application (see methods) to assign each point independently to each map class, without reference to the classification results or with each other.

The standard remote sensing error matrix approach was performed using standard methods^59^, with the mode of the three independently annotated validation sets representing the validated class. To align with reference imagery in the online application, map classes in the validation set were sampled from the version 1.1 2014–16 map. The initial error matrix analysis suggested overall map accuracy was 82.3% (Table 2) and the bootstrapped estimate of map accuracy, performed with 1,000 iterations^1^ suggested an overall map accuracy of 82.2% (80.0–84.3%, 95% confidence interval). To perform the third validation, an assessment of agreement with an independently produced map of intertidal extent for Australia from Landsat Archive data^61^, we randomly sampled the Australian map at 4,000 location across the Australian coastline with stratification over the two map classes (‘intertidal’ and ‘other’). The results indicated an 84.6% agreement between the two map products, with disagreement primarily attributed to the different periods mapped by each project (Australia, 1987–2016, global tidal flats, 2014–16) and potentially different definitions of target map classes^1^.

**Table 2:** Confusion matrix for the 2016 tidal flat distribution data (version 1.1).

		Reference		User’s (%)
		Other	Tidal Flat	
Classified	Other	566	113	83.4
	Tidal flat	128	551	81.1
Producer’s (%)		81.6	83.0	
Overall accuracy				82.3

The validated class is the mode of the three independently annotated validation sets collected for the 2014–2016 reference period (n = 1,358 samples). Table reproduced from^1^; refer to that publication for full accuracy assessment results (version 1.1).

To validate the version 1.2 global tidal flat data, we used the multi-analyst validation set (n = 1,358 random samples) to sample the 2014–16 map and perform the using standard accuracy assessment methods^54^ described above. The initial error matrix using the mode of the three analyst annotations suggested an overall map accuracy of 86.1% (Table 3). The bootstrapped estimate of overall map accuracy was 86.1% (84.2–86.8%, 95% confidence interval).

**Table 3:** Confusion matrix for the 2016 tidal flat distribution data (version 1.2).

		Reference		User’s (%)
		Other	Tidal Flat	
Classified	Other	621	116	84.3
	Tidal flat	73	548	88.3
Producer’s (%)		89.5	82.5	
Overall accuracy				86.1

The validated class is the mode of the three independently annotated validation sets collected for the 2014–2016 reference period (n = 1,358 samples).

Reference to imagery suggests that most classification errors in the tidal flat map class were due to the presence of highly turbid water, polluted waterways, seasonal sea ice and aquaculture^1^. Due to a lack of historical high-resolution imagery sufficient for use in validating the historical time-series maps, map validation was conducted only for the 2014–16 global map (version 1.1 and version 1.2). Refer to Usage Notes for further information.

## Usage Notes

In addition to a wide range of already published examples^1–3,40–47^, we expect the global tidal flat map data will support a range of efforts to investigate coastal environments at the global scale. Rapid migration of humans to coastal environments over the last few decades necessitate a detailed understanding of growing pressures on coastal ecosystems^62,63^, and time-series spatial data can support investigations of how natural ecosystems are responding to these pressures. In addition, integrating our fine-scale data of intertidal coastal zones into topographic or bathymetric models can improve our understanding of the observed and expected effects of sea level rise on factors such as tidal dynamics and coastal sediment dynamics^8,64^. The dataset is also likely to support growing efforts to assess risks of ecosystem collapse of a variety of coastal ecosystem types under the IUCN Red List of Ecosystems categories and criteria^12,65,66^.

By providing an empirical understanding of the distribution of tidal flat ecosystems, we also expect the global tidal flat data to improve simulation models that aim to estimate the future distribution of coastal environments at the global scale^67^. Such models must account for dynamic transitions in intertidal ecosystem types in response to changing sea level across the full land-sea interface. If combined with map data of other coastal ecosystems^8,27,68^, our data allows an incremental step towards synoptic analyses of dynamic intertidal ecosystem transitions at the global scale.

However, there are several important considerations regarding use of the global tidal flat map data:

1. Map coverage. Owing to the seasonal presence of sea ice and limitations in the use of Landsat sensor data to image high arctic regions^1,69^, the tidal flat maps do not include tidal flats that occur north of 60°N or south of 60°S.
2. Time-series coverage. Consistent time-series data is only available for a subset of the mapped area because Landsat archive data has not been consistently available for all areas of the world’s coastline^1^. Furthermore, when version 1.1 of the data was run, the entire Landsat Archive had not been fully ingested into Earth Engine. Thus, some areas, such as North America, East Asia and the Middle East, had more data available in each time period to compute predictor layers^1^. As noted in Murray *et al*.^1^, time-series of the map data should be developed using the quality assurance layers, which depict the number of pixels used to develop the 54 Landsat composite metric layers, to ensure sufficient imagery to reliably compute the predictors representing pixel-scale variance. Note version 1.2 improves the coverage of the world where globally consistent time-series can be developed but, owing to resource limitations, has been run only for the period 1999–2019. Users should expect the next update in 2023 (version 1.3).
3. Accuracy. Although the accuracy assessment indicated 82.3% (version 1.1) and 86.1% (version 1.2) overall accuracy when compared to validation data, there are unavoidable commission and omission mapping errors in the dataset. In many coastal areas, other land cover types such as aquaculture and rice agriculture undergo a similar wetting and drying regime as tidal flat pixels, resulting in mapping commission error. In addition, areas where there are few satellite images available or where waterbodies are highly turbid can result in commission error. Lastly, some areas where significant coastal change has occurred within each 3-year time period, such as rapid coastal development, have been incorrectly mapped as tidal flat. We therefore recommend statistical analysis approaches that account for variable measurement error^1,35^, and encourage users to provide feedback for incorporation in future releases (https://www.intertidal.app/contribute).
4. Comparisons with publicly available data. Our dataset was the first to map tidal flat ecosystems at the global scale. Since the open-access publication of the data^1^, several regional scale analyses have been conducted to advance the field of tidal flat remote sensing, which offers the opportunity to compare our methods, designed to meet design criteria including worldwide spatial consistency and an ability to implement in Google Earth Engine. These studies suggest that, despite differing study aims, the global tidal flat data showed an overall average agreement of >93% with tidal flat maps for the USA^70^ and ‘good agreement’ with newly developed tidal wetland maps developed for China^71^. The global tidal flat data also supported the development of 10-m spatial resolution maps of the intertidal zone for United Kingdom and Republic of Ireland^72^. Two studies^63,71^ reported that the global tidal flat data overestimated tidal flat extent in Asia due to commission error with coastal aquaculture. However, as noted in User Note (3), we discourage directly estimating tidal flat extent from our dataset without propagating known data uncertainties and reporting uncertainty estimates.
5. Observed tidal flat extent. It is unlikely that the full extent of tidal flats is acquired during satellite image acquisition. Therefore, the dataset should be considered ‘observed tidal flat extent’ ^1,35^.
6. Differences between Version 1.1 and Version 1.2. Relative to the version 1.1 map product, several modifications have been made:
  - A shorter study overall time-period. The shorter period (1999–2019) due primarily to resource limitations and significantly higher numbers of Landsat images requiring processing.
  - An additional time step. A new time step (2017–2019) is available in version 1.2.
  - Different mapped areas. We further reduced the area for which each 30-m pixel was subject to classification in version 1.2 to reduce computing time and enable saving of covariate data (which were produced and discarded on-the-fly for version 1.1).
  - Transition to Landsat Collection data. Version 1.2 uses Landsat Collection-1 data, whereas version 1.1 used a deprecated surface reflectance product that was formerly available in Google Earth Engine. Use of Landsat Collection-1 additionally allowed the per-pixel masking of snow and ice during development of the Landsat covariates, which was not done in version 1.1, yielding improvements to overall map accuracy (Table 3).
  - Expanded area where consistent time-series can be produced. Owing to increased numbers of Landsat images from the Landsat Archive available in Google Earth Engine between version 1.1 and version 1.2, the area for which consistent time-series can be produced has been greatly expanded. Refer to Usage Notes point (2) for recommendations on how to use the quality assurance layers to develop consistent time-series.
  - Expansion of the training set. The version 1.2 classification model uses additional training data that was targeted in areas where there was classification error in version 1.1.
  - Model parameters. The number of trees evaluated in the Random Forest classification model was changed from n = 10 (version 1.1) to n = 100 (version 1.2).
  - The post-processing mask to remove obvious misclassifications was updated.

It should be noted that the majority of these changes were implemented to promote data freshness^73^ of the tidal flat data (by adding the additional time-step).

## Data Availability

The Google Earth Engine JavaScript code to develop covariate layers and estimate the distribution of tidal flats globally is archived on Zenodo^58^.
